# Mobile Assessment of Acute Effects of Marijuana on Cognitive Functioning in Young Adults: Observational Study

**DOI:** 10.2196/16240

**Published:** 2020-03-10

**Authors:** Tammy Chung, Sang Won Bae, Eun-Young Mun, Brian Suffoletto, Yuuki Nishiyama, Serim Jang, Anind K Dey

**Affiliations:** 1 Department of Psychiatry University of Pittsburgh School of Medicine Pittsburgh, PA United States; 2 School of Systems and Enterprises Stevens Institute of Technology Hoboken, NJ United States; 3 Department of Health Behavior and Health Systems University of North Texas Health Science Center Fort Worth, TX United States; 4 Department of Emergency Medicine University of Pittsburgh School of Medicine Pittsburgh, PA United States; 5 Institute of Industrial Science The University of Tokyo Tokyo Japan; 6 Dietrich College of Humanities and Social Sciences Carnegie Mellon University Pittsburgh, PA United States; 7 Information School University of Washington Seattle, WA United States

**Keywords:** marijuana, cannabis, cell phone, memory, short-term, cognition

## Abstract

**Background:**

Mobile assessment of the effects of acute marijuana on cognitive functioning in the natural environment would provide an ecologically valid measure of the impacts of marijuana use on daily functioning.

**Objective:**

This study aimed to examine the association of reported acute subjective marijuana high (rated 0-10) with performance on 3 mobile cognitive tasks measuring visuospatial working memory (Flowers task), attentional bias to marijuana-related cues (marijuana Stroop), and information processing and psychomotor speed (digit symbol substitution task [DSST]). The effect of distraction as a moderator of the association between the rating of subjective marijuana high and task performance (ie, reaction time and number of correct responses) was explored.

**Methods:**

Young adults (aged 18-25 years; 37/60, 62% female) who reported marijuana use at least twice per week were recruited through advertisements and a participant registry in Pittsburgh, Pennsylvania. Phone surveys and mobile cognitive tasks were delivered 3 times per day and were self-initiated when starting marijuana use. Completion of phone surveys triggered the delivery of cognitive tasks. Participants completed up to 30 days of daily data collection. Multilevel models examined associations between ratings of subjective marijuana high (rated 0-10) and performance on each cognitive task (reaction time and number of correct responses) and tested the number of distractions (rated 0-4) during the mobile task session as a moderator of the association between ratings of subjective marijuana high and task performance.

**Results:**

Participants provided 2703 data points, representing 451 reports (451/2703, 16.7%) of marijuana use. Consistent with slight impairing effects of acute marijuana use, an increase in the average rating of subjective marijuana high was associated with slower average reaction time on all 3 tasks—Flowers (B=2.29; SE 0.86; *P*=.008), marijuana Stroop (B=2.74; SE 1.09; *P*=.01), and DSST (B=3.08; SE 1.41; *P*=.03)—and with fewer correct responses for Flowers (B=−0.03; SE 0.01; *P*=.01) and DSST (B=−0.18; SE 0.07; *P*=.01), but not marijuana Stroop (*P*=.45). Results for distraction as a moderator were statistically significant only for certain cognitive tasks and outcomes. Specifically, as hypothesized, a person’s average number of reported distractions moderated the association of the average rating of subjective marijuana high (over and above a session’s rating) with the reaction time for marijuana Stroop (B=−52.93; SE 19.38; *P*=.006) and DSST (B=−109.72; SE 42.50; *P*=.01) and the number of correct responses for marijuana Stroop (B=−0.22; SE 0.10; *P*=.02) and DSST (B=4.62; SE 1.81; *P*=.01).

**Conclusions:**

Young adults’ performance on mobile cognitive tasks in the natural environment was associated with ratings of acute subjective marijuana high, consistent with slight decreases in cognitive functioning. Monitoring cognitive functioning in real time in the natural environment holds promise for providing immediate feedback to guide personal decision making.

## Introduction

### Background

Adverse effects of marijuana use on cognitive functioning have been reported by some young adults [1,2], with associated negative consequences such as injury and fatality due to driving while *high* on marijuana [3,4]. The emerging research base on cognitive impairments associated with marijuana use indicates slight and selective cognitive functioning impairments, particularly with certain early onset, heavy, and chronic patterns of marijuana use [5,6]. The effects of marijuana use on cognition, in particular, also depend on factors such as the cognitive domain and whether testing occurs during acute (within 0-6 hours of use) or nonacute (>6 hours since use) periods. Although cross-sectional laboratory studies have compared individuals who use marijuana with healthy controls on measures of cognitive functioning [6], much less is known about the acute effects of marijuana use on cognitive functioning in the natural environment.

### Acute Effects of Marijuana on Cognition in Laboratory Studies

For smoked marijuana, the subjective effect of *feeling high* typically begins within 5 min of use and reaches a peak within 30 min, depending on the dose and smoking rate [7]. Laboratory studies indicate that during acute marijuana intoxication, verbal and working memory are typically impaired, and inhibitory control is reduced [8-10]. Findings from these laboratory studies [8-10] on the acute effects of marijuana on cognitive functioning guided the selection of the mobile cognitive tasks used in this study, which assess visuospatial working memory (Flowers task) [11], attentional bias to marijuana-related cues (marijuana Stroop) [12], and information processing and psychomotor speed (digit symbol substitution task [DSST]) [13,14].

### Mobile Cognitive Assessment Studies

To date, neuropsychological tests administered in laboratory settings have shown moderate associations with measures of daily functioning [15]. By comparison, data on cognitive functioning collected using ecological momentary assessment (EMA) have greater ecological validity than laboratory assessment as cognitive processes are assessed in real-world contexts [16]. A systematic review of mobile cognitive assessments reported good internal consistency and test-retest reliability for the mobile cognitive assessments studied, in addition to good convergent and divergent validity with laboratory-based measures [17]. The cognitive domain examined most often (7 studies) by mobile assessment was working memory [17]. For example, a 1-week EMA study that administered a mobile visual working memory task multiple (approximately 5-7) times per day to young adult cigarette smokers found that working memory performance decreased with acute marijuana (odds ratio [OR] 0.91, 95% CI 0.84-0.99) and alcohol use (OR 0.87, 95% CI 0.79-0.95) and increased with acute tobacco use (OR 1.11, 95% CI 1.04-1.18) [18]. Although this EMA study provides important insights into acute effects of substance use on working memory in the natural environment, the study focused on young adults who primarily reported cigarette rather than marijuana use; examined only working memory; and included limited information on the level of subjective marijuana high (ie, only examined yes or no reports of use) associated with working memory performance.

Another popular mobile cognitive task uses some version of an addiction Stroop [19]. The addiction Stroop measures attentional bias, the ability of substance-related stimuli to engage attention, particularly among individuals with heavier patterns of substance use [20]. For individuals with cannabis use disorders, cognitive biases for marijuana cues have generally been observed using different methods (eg, visual dot probe, marijuana Stroop) [19]. In a laboratory study examining marijuana Stroop, attentional bias was correlated with both the frequency of marijuana use and subjective craving [12]. Notably, no study to our knowledge has yet reported results for a mobile version of marijuana Stroop. In a mobile version of the alcohol Stroop, attentional bias scores were not associated with individual differences in drinking behavior [21]. In contrast, attentional bias for cigarette smoking cues assessed by a mobile version of the smoking Stroop administered on a personal digital assistant was associated with nicotine dependence severity [22] and nicotine craving during the early stages of a quit attempt [23]. The mixed findings for attentional bias assessed by mobile versions of an addiction Stroop might be due to factors such as the type of substance (alcohol and nicotine) assessed [20] and task parameters (eg, number of trials used in the task).

Two other pilot studies used mobile versions of a Stroop task. One study of outpatients with substance use disorders and healthy controls found practice effects with mobile tasks completed 5 times per day for a week, but only for healthy controls [24]. However, another study that examined a mobile Stroop found no practice effects among participants with methamphetamine dependence or healthy controls who completed mobile tasks twice daily for 2 weeks [25]. Another factor to consider in mobile cognitive assessment is the impact of distraction on task performance [23]. On a cigarette smoking Stroop task, the number of reported interruptions during task performance was associated with slower reaction times and more errors, but interruptions were not associated with the cigarette smoking Stroop effect (ie, slow reaction time when viewing smoking-related words) [23]. These findings suggest the use of examining distraction as a moderator of mobile task performance, in addition to considering practice effects.

### Study Objectives

Informed by laboratory research, this pilot EMA study explored the acute effects of marijuana use on young adults’ performance during 3 brief mobile cognitive tasks assessing visuospatial working memory (Flowers task), attentional bias to marijuana-related words (marijuana Stroop), and information processing and psychomotor speed (DSST). Multilevel analyses, conducted separately for each of the 3 cognitive tasks and 2 outcomes (reaction time and number of correct responses), tested the hypothesis that as the rating of momentary subjective marijuana high increases, the reaction time on the mobile cognitive task will slow down and the number of correct responses will decrease. Analyses also examined typical levels of distraction across sessions as a moderator of the association between the typical ratings of subjective marijuana high across sessions and cognitive task performance. Moderation analyses tested the hypothesis that the average number of reported distractions (across sessions) will intensify the effect of being high on marijuana on reaction time and the number of correct responses.

## Methods

### Recruitment

Young adults (aged 18-25 years) who reported marijuana use at least two times per week (44/71, 62% female) were recruited through a participant registry (Pitt+Me) and Craigslist advertisements in Pittsburgh, Pennsylvania. The exclusion criteria were as follows: currently seeking treatment for substance use, self-reported history of psychosis, and use of medication or a device (eg, pacemaker) that could affect the heart rate.

### Participants

Individuals who completed at least five mobile sessions (1 mobile session=1 phone survey + 3 cognitive tasks) were included in the analyses, based on research suggesting that participants gain familiarity with mobile tasks during early sessions (ie, first 5 sessions) [26]. Completion of the phone survey immediately triggered the cognitive tasks. Participants who did not complete at least five sessions (n=3) were excluded. Participants who had scores only when high on marijuana (n=4) were excluded as they do not provide information on session-level comparisons of *high* vs *not high* on marijuana. In addition, 4 participants with missing scores for estimated intellectual functioning (see *Baseline Measures*) were excluded from the analyses. Thus, the analysis sample included 60 participants, of which 37 (62%) were female (mean age 20.0, SD 1.8 years), 45 (75%) were white, 8 (13%) were black, and 7 (12%) were of another race or ethnicity (ie, Asian, Asian Indian, Hispanic, or multiracial). Most participants (40/60, 66%) reported attending some college, 25% (15/60) reported having a high school diploma or equivalent, and 8% (5/60) were college graduates. The majority (55/60, 92%) owned an iOS device, and 8% (5/60) owned an Android mobile phone.

### Procedure

Eligible individuals provided written informed consent for study participation. At the baseline assessment, participants installed study mobile apps (eg, AWARE [27] to deliver phone surveys, MUSE [28] to deliver cognitive tasks) on their personal phones, and research staff trained participants on completion of the mobile surveys and cognitive tasks. At baseline, participants completed an interview and questionnaires assessing demographic characteristics, substance use history, and neuropsychological measures assessing attention, memory, and response inhibition. IQ was estimated using a reading test [29]. After baseline, participants completed up to 30 days of daily data collection (see *Compensation*). Daily data collection included scheduled assessments and user-initiated assessments (see *Phone Surveys: Self-Initiated Marijuana Use and Fixed Time Daily Surveys*). At the end of the daily data collection period (phone surveys and mobile cognitive tasks), participants completed a wrap-up session. The University of Pittsburgh institutional review board approved the research protocol.

### Baseline Measures

The National Institute on Drug Abuse (NIDA) Quick Screen [30] is a widely used measure to screen 10 types of substance use and substance-related problems covering time frames of lifetime (yes or no) and past 3 months (5-point scale: 0=never to 4=almost daily). Scores of 0 to 3 indicate low risk, 4 to 26 indicate moderate risk, and ≥27 indicate high risk.

The National Adult Reading Test-Revised [29] provided an estimate of full-scale IQ (FSIQ) to account for individual differences in premorbid IQ, which might affect cognitive task performance [10]. A validation study found that National Adult Reading Test FSIQ estimates were similar to Wechsler Adult Intelligence Scale-Revised estimates [31]. The sample mean estimated FSIQ was 110.9 (SD 6.1; range 89.9-121.6).

### Phone Surveys: Self-Initiated Marijuana Use and Fixed Time Daily Surveys

Completion of the self-initiated and fixed time daily surveys both immediately triggered the start of mobile cognitive tasks. Participants were instructed to complete self-initiated reports at the start of marijuana use (ie, typically within the first 15 min of initiating use, *when feeling high*). Participants reported the time marijuana use started, mode of use (eg, joint, vape, pen, and bowl), quantity consumed (eg, grams or hits), on the question “How high are you feeling right now?” (0=none to 10=a lot), and other substance use (eg, number of drinks consumed).

Fixed-time daily surveys were delivered 3 times per day (ie, 10 AM, 3 PM, and 8 PM) with a 5-hour window for completion. Participants received a notification that the survey and tasks were available but did not receive reminders to complete the survey. Fixed-time surveys (similar to self-initiated reports) included items on time of last marijuana use, quantity consumed, the question “How high are you feeling right now?” and other items (eg, mood rating). Survey completion immediately triggered the administration of the 3 cognitive tasks in a randomized order. A session (a phone survey and mobile cognitive tasks) *timed out* if there was a lag in response for >1 min, which would end the session, such that remaining tasks and post-task survey items (eg, distraction item, see below) could not be done. With this schedule of fixed time and self-initiated assessments, participants reported their rating of subjective marijuana high immediately before performing the mobile cognitive tasks, which permitted the examination of task performance when participants reported not being high (subjective high rating=0) relative to reports when feeling *high* (subjective high rating>0).

### Mobile Phone Cognitive Tasks and Rating of Distraction After Session Completion

The 3 brief cognitive tasks (approximately 5 min in total to complete) included the following: visuospatial working memory task (Flowers task [11]), marijuana Stroop [12], and DSST [13]. The Flowers task and marijuana Stroop provided immediate feedback on incorrect responses. The DSST did not provide any feedback on correct or incorrect responses to minimize distractions during task performance. The 3 tasks did not provide a score regarding performance.

**Figure 1:**
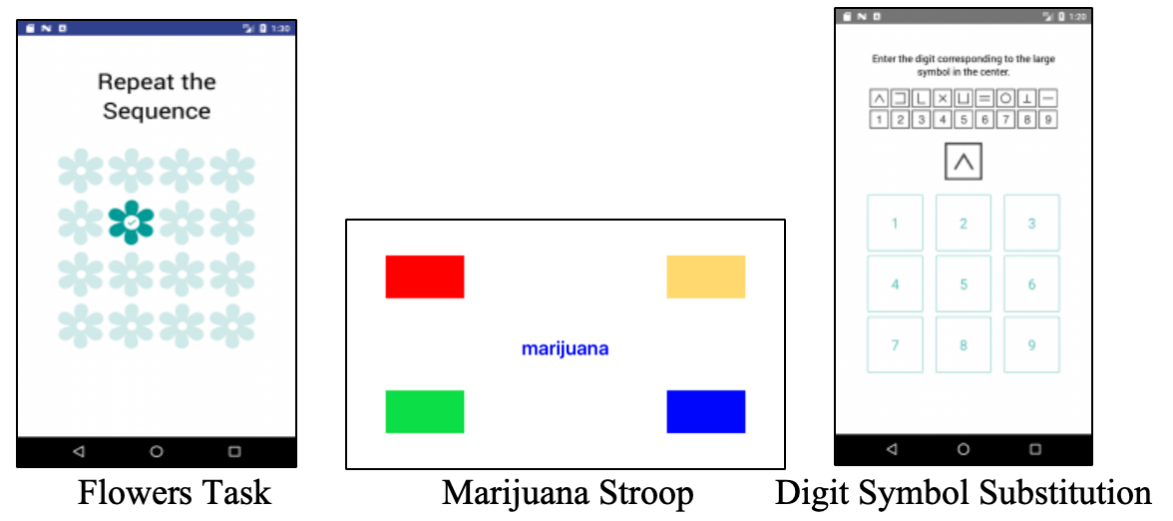
Screenshots of Flowers task, marijuana Stroop, and digit symbol substitution task.

The Flowers task assesses short-term visuospatial working memory [11]. Participants watched flowers in a grid light up one at a time and were instructed to replicate the sequence by touching the flowers in the grid in the same order (Figure 1). The task adapts to a test taker’s ability and increases or decreases its difficulty, starting with a 3×3 grid and increasing to a 4×4 grid with success or decreasing in difficulty with error. The task ends with 2 consecutive errors or a maximum of 6 correct responses. Previous work found that task performance (ie, number of correct responses) distinguished patients with Parkinson disease from healthy controls [26]. The Flowers task scores are the number of correct responses [11] and reaction time. Reaction time was added to assess possible psychomotor slowing associated with acute marijuana use, similar to the other 2 mobile tasks. Embedded sensors in mobile phones allow precise measurement for reaction time tasks [32].

Marijuana Stroop [12] measures attentional bias for marijuana-related stimuli. Participants were presented with 14 marijuana-related words (eg, hash and joint) and 14 neutral words (eg, sand and winds) in a randomized order in 2 blocks. Words appeared in 4 colors (red, yellow, blue, and green). Word color was random, but each color was shown at least once in each set of 28 words. Participants tapped the color of the word as fast as possible (Figure 1). Errors were shown by a red X immediately after the response. A computerized version of the marijuana Stroop task indicated that marijuana-related words captured the attention of marijuana-dependent individuals (ie, longer reaction time for marijuana-related words vs neutral words), but not healthy controls [12]. A mobile alcohol Stroop task had acceptable internal consistency reliability in real-world settings (Cronbach alpha=.70 to .74), with participants showing attentional bias to alcohol words [21].

To compute the marijuana Stroop reaction time score, trials with incorrect responses were excluded, and trials with unrealistically fast (<100 ms; none excluded) or slow (>1250 ms) reaction times were excluded (88/1467, 6.00% of total responses) [33,34]. Internal consistency reliability (Cronbach alpha) for response latency was computed by averaging within each trial type [34] for marijuana-related words and neutral words (alpha=.92 and .92, respectively). The total number of correct responses in the marijuana Stroop task also excluded trials with unrealistically fast or slow reaction times. There were no significant results from multilevel analyses for a marijuana Stroop effect (results not shown), computed as the difference in reaction time for marijuana-related and neutral words [12]. Preliminary multilevel analyses, which examined marijuana Stroop reaction time scores for combined marijuana and neutral words and separately by word type (marijuana-related and neutral) [12], indicated similar results for combined and separate word types. Similarly, preliminary multilevel results for the number of correct responses were similar for a combination of marijuana-related and neutral words and separate word types. Thus, marijuana Stroop scores are reaction time and the total number of correct responses (a combination of marijuana-related and neutral words).

DSST [13] measures information processing and psychomotor processing speed and is sensitive to acute drug effects [14]. The DSST requires quick response to visual symbols by touching the corresponding digit (1-9) shown in the reference key (Figure 1). New reference keys provided after each response minimize learning effects within the 60-second task session. No feedback was provided regarding a correct or an incorrect response. DSST scores are reaction time and the number of correct responses.

The number of distractions while performing the cognitive tasks was reported after completing the 3 tasks by responding to the following item: “How many times were you distracted during completion of the tasks?” (coded 0 to 4 or more times; 0-4) [23].

### Compensation

Participants were compensated US $75 for completing the baseline assessment. During the first 14 days of daily data collection, for each day on which >75% of data were collected (eg, phone surveys and cognitive tasks), participants earned US $10; if <75% of data were collected on a particular day, no money was earned. If the participant had good compliance during the first 14 days of data collection and was willing to continue for another 14 days, data collection continued for a second 14-day period at the same compensation rate. Participants earned US $25 for the wrap-up session (user experience interview and final data download).

### Statistical Analysis

To examine the acute effects of marijuana use on mobile cognitive task performance, generalized linear mixed effects models were fit to the data (xtmixed: Stata Statistical Software 15.0, StataCorp LLC) [35]. This multilevel modeling approach can accommodate mixed (fixed and random) effects across multiple data levels and account for the correlations between repeated measures (ie, sessions: level 1) within participants (level 2). Mixed models used maximum likelihood estimation, leveraging all available data to accommodate missing data. Likelihood ratio testing (−2LL difference between models) evaluated the statistical significance of nested models when random effects were added, and Akaike and Bayesian Information Criteria were used to evaluate the model fit between non-nested models [36].

The main outcomes of reaction time and number of correct responses were examined for each of the 3 mobile cognitive tasks. Distributions for the outcome variables of the number of correct responses for the Flowers and marijuana Stroop tasks indicated negative skew. Exponential (cubic) transformation reduced skew but did not normalize distributions and resulted in similar findings. Thus, untransformed results are reported. Analyses used the first 60 completed sessions (sparse data at >60 sessions). Alpha was set at *P*<.05 without protecting the family-wise alpha rate for multiplicity in this pilot study.

**Figure 2:**
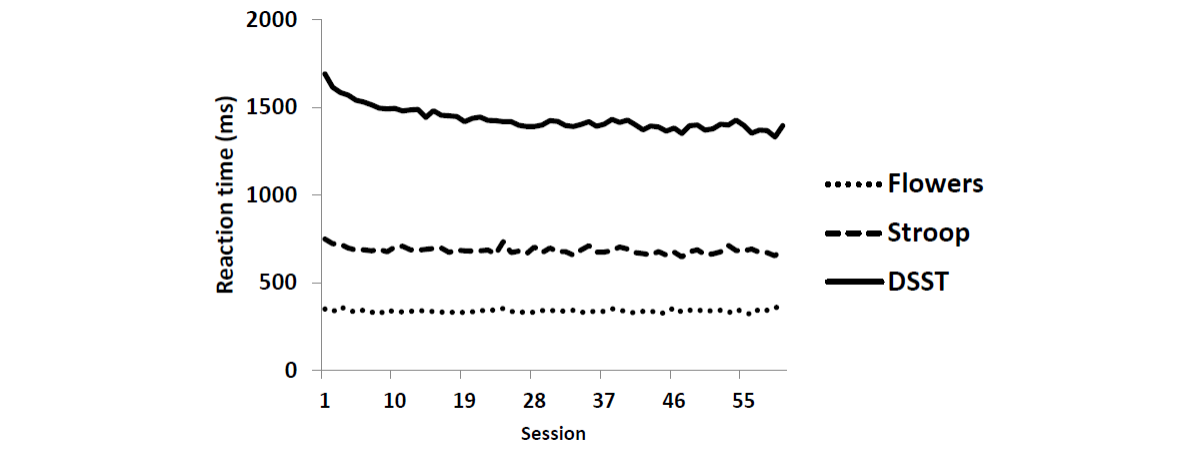
Average reaction time across sessions: flowers, marijuana Stroop, and digit symbol substitution task tasks. DSST: digit symbol substitution task.

**Figure 3:**
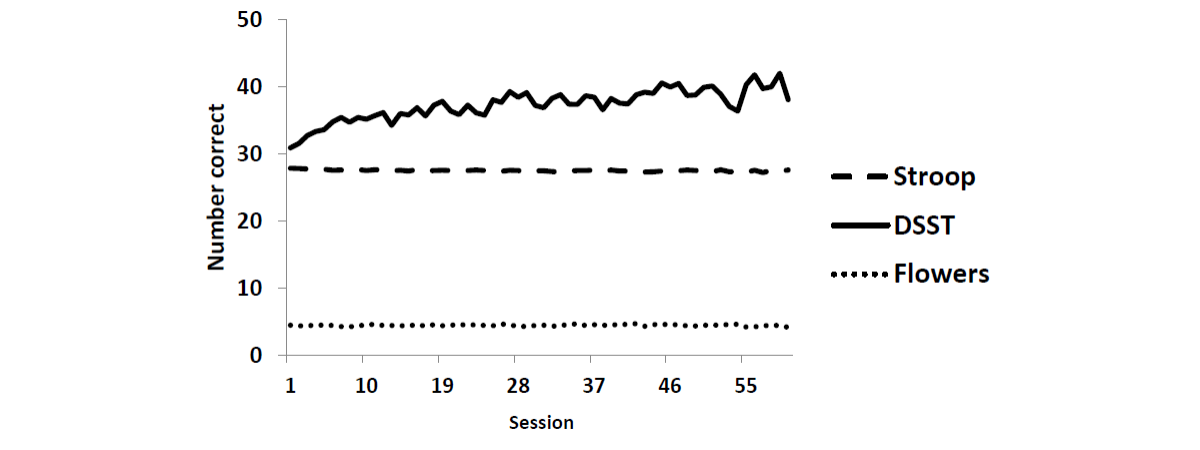
Average number of correct responses across sessions: flowers, marijuana Stroop, and digit symbol substitution task tasks. DSST: digit symbol substitution task.

Preliminary analyses examined correlations, computed intraclass correlations (ICCs), and modeled time trends (ie, practice effects; see Figures 2 and 3) for the outcomes (reaction time and number of correct responses). ICCs and time trends were examined for time-varying predictors (eg, subjective marijuana high and distraction) in unconditional models. As experience with the cognitive task itself (rather than the passage of time) likely contributes to a *practice* effect, the sequential count of completed sessions was used as the measure of time [36]. Time was coded so that the first completed session (done at baseline) represented session=0 in all models. For both outcomes, the model that provided the best fit was a linear model for Flowers and marijuana Stroop tasks and a quadratic model for DSST.

In total, 2 time-varying predictors were examined: subjective marijuana high (“How high are you feeling right now?” rated 0-10) and distraction (“How many times were you distracted during completion of the tasks?” coded 0-4) and their interaction. Decomposition of person-level (level 2) and session-level (level 1) effects in the mixed model was done as follows: the time-varying covariates (eg, subjective marijuana high and distraction) were centered at a constant (ie, 0; *constant-centered [CC]*) and tested in the model as level 1 predictors; and the corresponding person mean counterpart was entered simultaneously at level 2 [36]. When the CC and person mean–centered variables are entered together in the model, the CC variable represents session-to-session variation. The person mean variable represents the unique effect of the person’s average level of that variable on the outcome over and above the *absolute amount* of the time-varying CC effect, or in other words, individual differences in subjective marijuana high or distraction across all sessions [36].

Subjective marijuana high was centered such that a rating of 5=*moderately high* was recoded to be centered at 0 (new range −5 to 5). Ratings of subjective high had an ICC of 0.04 and showed no systematic change over time. Ratings for distraction were not transformed (range 0-4; ICC=0.25) and were *centered* at 0, and also showed no systematic change over time. Person means for subjective marijuana high (referred to as *subjective high person mean*) and distraction (*distraction person mean*) were computed.

Static covariates entered at the person level (level 2) included gender (0=male and 1=female), age (0=age 20), and FSIQ (0=estimated FSIQ of 110). Interactions of time-varying predictors (eg, subjective marijuana high, distraction) with static covariates were not tested to limit multiple comparisons and because no *a priori* hypotheses for these interactions were proposed. Other drug use was explored for inclusion as a time-varying predictor but was highly collinear with a rating of subjective marijuana high and was not included (334/451, 74.0% of phone surveys reported no alcohol use; 408/451, 90.5% reported no nicotine use). Weekend or weekday use (see Multimedia Appendix 1) was not a significant predictor.

Likelihood ratio testing, which examined relative model fit when including random slopes for time and time-varying covariates (eg, subjective marijuana high and distraction), indicated that including random slopes for a session and subjective marijuana high did not improve the model fit. A random slope for distraction for the outcome of reaction time improved the model fit (see Multimedia Appendix 1) and was included in models for this outcome.

## Results

### Marijuana Use in the Sample

In the analysis sample, the average age of onset for marijuana use was 16.5 years (SD 1.7; range 13-22), and the average age of onset for regular marijuana use (ie, using marijuana at least once per month for at least six months) was 17.2 years (SD 1.6). At baseline, 27% (16/60) of participants reported daily marijuana use, 10% (6/60) reported *almost daily* use (5-6 times per week), 33% (20/60) reported use 3 to 4 times per week, and 30% (18/60) reported use 2 times per week. The mean score on the NIDA Quick Screen was 15.5 (SD 6.4) [30], indicating moderate risk associated with marijuana use. Almost all (54/57, 95%) participants scored in the moderate risk range, with the remaining (6/120, 5%) participants scoring in the severe range.

Across 60 sessions completed over 5 to 30 days (mean 20.6 days, SD 6.5; Multimedia Appendix 2), 2703 data points were obtained from 60 young adults who provided 451 reports (451/2703, 16.7%) of marijuana use. Reports were obtained from morning (133/451, 29.4%), afternoon (115/451, 25.6%), evening (106/451, 23.4%), and self-initiated marijuana surveys (97/451, 21.6%). The average number of sessions completed per participant was 25.3 (SD 16.2; Multimedia Appendix 3). Participants completed 52.98% (2119/4000) of fixed time assessments.

The average number of sessions associated with marijuana use was 7.0 (SD 5.8). During sessions completed when high on marijuana (*any* rating of feeling *high*; n=451), the average level of subjective marijuana high was rated 4.7 out of 10 (SD 2.2; range 1-10). The most common method of consumption was pen or vaporizer (207/451, 45.9%), followed by bong (100/451, 22.2%), bowl or pipe (59/451, 13.0%), joint (38/451, 8.5%), blunt (38/451, 8.4%), edible (5/451, 1.1%), or tincture (4/451, 0.8%). The average quantity consumed per occasion was 0.8 g (SD 1.1), and when reported as hits, an average of 6.0 hits (SD 11.6). There was a small positive correlation (*r*=0.13; *P*=.001) between the number of hits reported and the rating of subjective marijuana high, but no statistically significant association between the number of grams reported and rating of subjective marijuana high (*r*=−0.03; *P*=.58).

The average number of distractions reported during a mobile cognitive task session when not high was 1.1 (SD 1.1; range 0-4), and when high was 0.9 distractions (SD 1.0; range 0-4). There was a very small negative correlation (*r*=−0.04; *P*=.046) between the number of distractions reported and the rating of subjective marijuana high.

### Mobile Cognitive Tasks

Tables 1 and 2 show the intercorrelations among the mobile tasks for reaction time and number of correct responses, based on a subjective marijuana high rating of 0 (*not high*) vs a rating from 1 to 10 (when feeling *high*). Reaction times were positively correlated for all 3 tasks (*r*=0.18 to 0.48; *P*=.001). The number of correct responses was positively correlated for Flowers and DSST (*r*=0.26 to 0.27; *P*=.001), negatively correlated for DSST and marijuana Stroop (*r*=−0.14 to −0.18; *P*=.001) and showed no association for marijuana Stroop and Flowers task (*P*>.36). Raw differences in reaction times when high vs not high on the tasks were small (<50 ms), as were differences in the number of correct responses, generally indicating slightly slower reaction times and slightly fewer correct responses when high on marijuana (vs not high).

For reaction time, when not high on marijuana (subjective high rating=0), ICCs were as follows: DSST=0.57, marijuana Stroop=0.23, and Flowers=0.38. When high on marijuana (subjective high rating>0), ICCs for reaction time were as follows: DSST=0.61, marijuana Stroop=0.25, and Flowers=0.29. The ICCs indicated that between 23% and 61% of the variances in reaction time for mobile tasks were between persons.

For number of correct responses, when not high on marijuana, ICCs were as follows: DSST=0.49, marijuana Stroop=0.13, and Flowers=0.15. When high on marijuana, ICCs for number of correct responses were as follows: DSST=0.50, marijuana Stroop=0.11, and Flowers=0.27. For number of correct responses, 11% to 50% of the variances in mobile task performance were between persons. The generally lower ICCs for marijuana Stroop and Flowers tasks (ie, reaction time and number of correct responses) suggested that their overall variance primarily reflects within-person, session-to-session fluctuations, providing the rationale for a multilevel analysis.

**Table 1:** Pearson correlations (r) and mean reaction time (millisecond) for 3 cognitive tasks (correlations do not take clustering of cases within individuals over time into account).

Measure	Not high (subjective high rating=0), n=2159 sessions		High (subjective high rating >0), n=389 sessions		Not high	High
	Flowers	Marijuana Stroop	Flowers	Marijuana Stroop	DSST^a^	DSST
Marijuana Stroop	0.20^b^	N/A^c^	0.34 ^b^	N/A	N/A	N/A
DSST	0.18^b^	0.36^b^	0.25^b^	0.48^b^	N/A	N/A
Reaction time, mean (SD)	335.80 (73.38)	682.08 (115.24)	354.87 (80.51)	713.60 (139.73)	1440.83 (170.59)	1488.75 (187.56)

^a^DSST: digit symbol substitution task.

^b^*P*<.001.

^c^Not applicable.

**Table 2:** Pearson correlations (r) and mean number of correct responses for 3 cognitive tasks (correlations do not take clustering of cases within individuals over time into account).

Measure		Not high (subjective high rating=0), n=2252 sessions		High (subjective high rating >0), n=451 sessions		Not high	High
		Flowers	Marijuana Stroop	Flowers	Marijuana Stroop	DSST^a^	DSST
Marijuana Stroop		0.02	N/A^b^	−0.02	N/A	N/A	N/A
DSST		0.26^c^	−0.14^c^	0.27^c^	−0.18^c^	N/A	N/A
**Correct responses**							
	Mean (SD)	4.49 (0.88)	27.52 (0.77)	4.31 (1.09)	27.51 (0.81)	37.03 (7.76)	35.44 (8.58)
	Median (SD)	5.00 (1.00)	28.00 (1.00)	5.00 (1.00)	28.00 (1.00)	38.00 (9.00)	37.00 (10.00)

^a^DSST: digit symbol substitution task.

^b^Not applicable.

^c^*P*<.001.

### Reaction Time: Associations With Subjective Marijuana High and Distraction

For all 3 mobile cognitive tasks, there was a significant session-level association of subjective marijuana high with reaction time (Tables 3-5), such that an increase in the rating of subjective marijuana high was associated with slower reaction time: Flowers task (B=2.29; SE 0.86; *P*=.008), marijuana Stroop (B=2.74; SE 1.09; *P*=.01), and DSST (B=3.08; SE 1.41; *P*=.03). In addition, there was a significant effect of subjective high person mean for both marijuana Stroop (B=77.78; SE 25.48; *P*=.002), and DSST (B=181.32; SE 55.83; *P*=.001), indicating an effect over and above that of a specific session for slower (ie, increasing) reaction time with a greater average rating of subjective marijuana high across sessions (ie, the person’s *usual* level of high, relative to other people with lower ratings).

The sample size of the Flowers task has 2 fewer cases compared with the other 2 tasks because of the late initiation of reaction time data collection due to programming delay.

**Figure 4:**
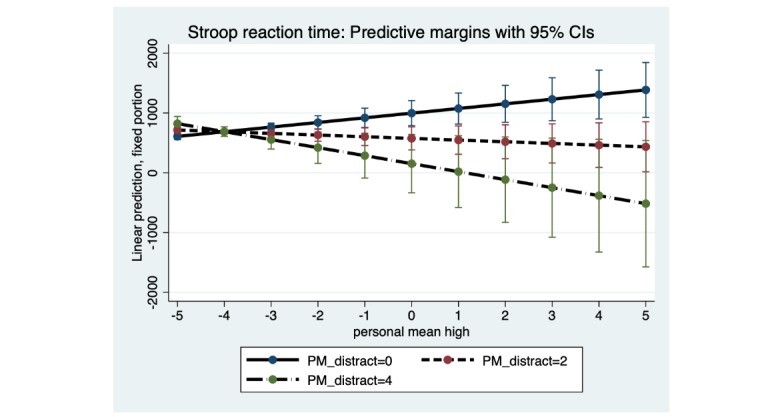
Marijuana Stroop: distraction person mean (PM) as a moderator of the association between subjective high PM and reaction time. Distraction PM at low (0), moderate (2) and high levels (4). PM: person mean.

**Figure 5:**
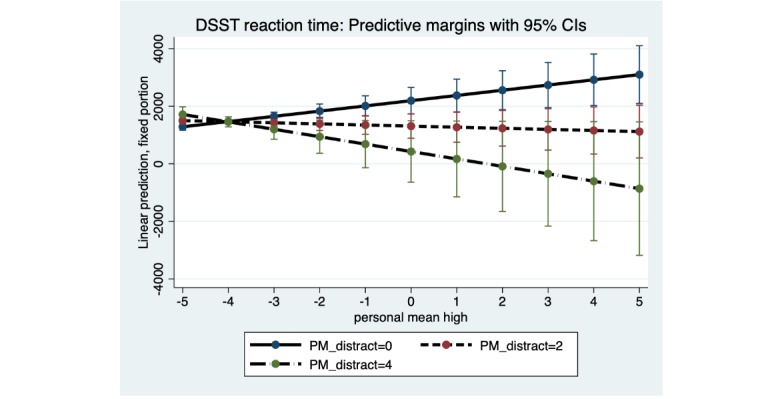
Digit symbol substitution task: distraction person mean (PM) as a moderator of the association between subjective high PM and reaction time. Distraction PM at low (0), moderate (2) and high levels (4). DSST: digit symbol substitution task; PM: person mean.

For both marijuana Stroop and DSST, there was a significant interaction of subjective marijuana high person mean with distraction person mean: marijuana Stroop (B=−52.92; SE 19.38; *P*=.006) and DSST (B=−109.72; SE 42.50; *P*=.01; Tables 4 and 5). For both tasks (Figures 4 and 5), there was little estimated difference in reaction time at low average levels of subjective marijuana high person mean, but contrary to the hypothesis, at higher average levels of distraction person mean and higher average ratings of subjective marijuana high person mean, reaction time was estimated to decrease (with wide 95% CIs at the highest average level of contextual distraction). In contrast, at low average levels of distraction person mean, reaction time was predicted to be slower as the subjective rating of high person mean increased (possibly reflecting the effect of marijuana on the slowing of psychomotor functioning).

**Table 3:** Flowers task: multilevel model of marijuana high in relation to reaction time.

Effects			Flowers reaction time (n=58)			
			Estimate	SE	95% CI	*P* value
**Fixed effects**						
	**Person level (level 2)**					
		Intercept	592.88	128.62	N/A^a^	.001^b^
		Session	−0.14	0.08	N/A	.08
		Session^2^ (quadratic)	N/A	N/A	N/A	N/A
		Subjective high (PM^c^)	57.45	30.16	N/A	.06
		Distraction (PM)	−81.80	89.75	N/A	.36
		Subjective high (PM) × distraction (PM)	−20.74	20.79	N/A	.32
		Gender (0=male, 1=female)	−9.64	10.59	N/A	.36
		Age (0=age 20)	6.49	3.17	N/A	.04^d^
		Full-scale IQ (0=IQ score of 110)	2.50	1.04	N/A	.02^d^
	**Session level (level 1)**					
		Subjective high (CC^e^)	2.29	0.86	N/A	.008^b^
		Distraction (CC)	−4.38	2.88	N/A	.13
		Subjective high (CC) × distraction (CC)	−0.39	0.55	N/A	.48
**Random effects**						
	Level 1 residual variance		3415.85	99.90	3225.55 to 3617.38	N/A
	Intercept		1259.15	274.92	820.79 to 1931.64	N/A
	Distraction		56.58	28.40	21.15 to 151.33	N/A
	Covariance (distraction, intercept)		0.48	71.70	−140.04 to 141.00	N/A

^a^Not applicable.

^b^*P*<.01.

^c^PM: person mean scores, reflecting individual differences in subjective marijuana high or distraction across all sessions.

^d^*P*<.05.

^e^CC: constant-centered scores (centered at 0), reflecting session-to-session variation in scores or session-specific scores.

**Table 4:** Marijuana Stroop: multilevel model of marijuana high in relation to reaction time.

Effects			Stroop reaction time (n=60)			
			Estimate	SE	95% CI	*P* value
**Fixed effects**						
	**Person level (level 2)**					
		Intercept	1018.27	106.73	N/A^a^	.001^b^
		Session	−0.61	0.10	N/A	.001^b^
		Session^2^ (quadratic)	N/A	N/A	N/A	N/A
		Subjective high (PM^c^)	77.78	25.48	N/A	.002^b^
		Distraction (PM)	−211.10	82.07	N/A	.01^d^
		Subjective high (PM) × distraction (PM)	−52.92	19.38	N/A	.006^b^
		Gender (0=male, 1=female)	2.40	14.31	N/A	.87
		Age (0=age 20)	12.78	4.08	N/A	.002^b^
		Full-scale IQ (0=IQ score of 110)	−0.03	1.38	N/A	.98
	**Session level (level 1)**					
		Subjective high (CC^e^)	2.74	1.09	N/A	.01^d^
		Distraction (CC)	−0.44	3.94	N/A	.91
		Subjective high (CC) × distraction (CC)	−10.32	0.72	N/A	.07
**Random effects**						
	Level 1 residual variance		5858.83	168.35	5537.99 to 6198.26	N/A
	Intercept		2647.94	574.67	1730.52 to 4051.72	N/A
	Distraction		188.00	67.35	93.16 to 379.40	N/A
	Covariance (distraction, intercept)		−162.67	158.49	−473.31 to 147.96	N/A

^a^Not applicable.

^b^*P*<.01.

^c^PM: person mean scores, reflecting individual differences in subjective marijuana high or distraction across all sessions.

^d^*P*<.05.

^e^CC: constant-centered scores (centered at 0), reflecting session-to-session variation in scores or session-specific scores.

**Table 5:** Digit symbol substitution task: Multilevel model of marijuana high in relation to reaction time.

Effects			DSST^a^ reaction time (n=60)			
			Estimate	SE	95% CI	*P* value
**Fixed effects**						
	**Person level (level 2)**					
		Intercept	2328.14	233.72	N/A^b^	.001^c^
		Session	−9.45	0.45	N/A	.001^c^
		Session^2^ (quadratic)	0.12	0.01	N/A	.001^c^
		Subjective high (PM^d^)	181.32	55.83	N/A	.001^c^
		Distraction (PM)	−441.54	179.82	N/A	.01^e^
		Subjective high (PM) × distraction (PM)	−109.72	42.5	N/A	.01^e^
		Gender (0=male, 1=female)	−2.75	31.61	N/A	.93
		Age (0=age 20)	7.55	8.92	N/A	.4
		Full-scale IQ (0=IQ score of 110)	4.91	3.02	N/A	.1
	**Session level (level 1)**					
		Subjective high (CC^f^)	3.08	1.41	N/A	.03^e^
		Distraction (CC)	2.21	4.6	N/A	.63
		Subjective high (CC) × distraction (CC)	−1.01	0.92	N/A	.27
**Random effects**						
	Level 1 residual variance		9950.65	285.83	9405.91 to 10,526.95	N/A
	Intercept		12906.21	2506.7	8820.12 to 18,885.27	N/A
	Distraction		63.55	59.06	10.28 to 392.83	N/A
	Covariance (distraction, intercept)		−67.68	330.49	−715.43 to 580.07	N/A

^a^DSST: digit symbol substitution task.

^b^Not applicable.

^c^*P*<.01.

^d^PM: person mean scores, reflecting individual differences in subjective marijuana high or distraction across all sessions.

^e^*P*<.05.

^f^CC: constant-centered scores (centered at 0), reflecting session-to-session variation in scores or session-specific scores.

### Number of Correct Responses: Associations With Subjective Marijuana High and Distraction

There was a significant session-level association of subjective marijuana high with the number of correct responses for Flowers (B=−0.03; SE 0.01; *P*=.01) and DSST (B=−0.18; SE 0.07; *P*=.01) tasks, such that the increase in the rating of subjective marijuana high was associated with fewer correct responses (eg, Table 6). Although the session-level effect of subjective marijuana high on number of correct responses for marijuana Stroop was not significant (*P*=.45; Table 7), the effect of subjective marijuana high person mean was statistically significant (B=0.37; SE 0.13; *P*=.003), indicating unique effects of the person’s average subjective rating of marijuana high across sessions controlling for a specific occasion’s rating of subjective marijuana high on the number of correct responses in the marijuana Stroop task. For DSST (Table 8), the effect of subjective marijuana high person mean was also significant (B=−6.83; SD 2.38, *P*=.004), indicating that at an average subjective rating of marijuana high, the number of correct DSST responses was lower (controlling for a specific occasion’s rating of subjective marijuana high).

**Table 6:** Flowers task: multilevel model of marijuana high in relation to number of correct responses.

Effects			Flower number of correct responses (n=58)			
			Estimate	SE	95% CI	*P* value
**Fixed effects**						
	**Person level (level 2)**					
		Intercept	2.92	0.85	N/A^a^	.001^b^
		Session	0	0	N/A	.007^b^
		Session^2^ (quadratic)	N/A	N/A	N/A	N/A
		Subjective high (PM^c^)	−0.35	0.2	N/A	.08
		Distraction (PM)	0.83	0.65	N/A	.2
		Subjective high (PM) × distraction (PM)	0.18	0.15	N/A	.23
		Gender (0=male, 1=female)	−0.05	0.11	N/A	.64
		Age (0=age 20)	−0.14	0.03	N/A	.001^b^
		Full-scale IQ (0=IQ score of 110)	0	0.01	N/A	.66
	**Session level (level 1)**					
		Subjective high (CC^d^)	−0.03	0.01	N/A	.01^e^
		Distraction (CC)	−0.13	0.03	N/A	.001^b^
		Subjective high (CC) × distraction (CC)	0.01	0.01	N/A	.21
**Random effects**						
	Level 1 residual variance		0.59	0.02	0.56 to 0.63	N/A
	Intercept		0.16	0.03	0.10 to 0.24	N/A

^a^Not applicable.

^b^*P*<.01.

^c^PM: person mean scores, reflecting individual differences in subjective marijuana high or distraction across all sessions.

^d^CC: constant-centered scores (centered at 0), reflecting session-to-session variation in scores or session-specific scores.

^e^*P*<.05.

**Table 7:** Marijuana Stroop task: multilevel model of marijuana high in relation to number of correct responses.

Effects			Stroop number of correct responses (n=60)			
			Estimate	SE	95% CI	*P* value
**Fixed effects**						
	**Person level (level 2)**					
		Intercept	29.03	0.53	N/A^a^	.001^b^
		Session	−0.00	0	N/A	.001^b^
		Session^2^ (quadratic)	N/A	N/A	N/A	N/A
		Subjective high (PM^c^)	0.37	0.13	N/A	.003^b^
		Distraction (PM)	−0.82	0.41	N/A	.046^d^
		Subjective high (PM) × distraction (PM)	−0.22	0.1	N/A	.02^d^
		Gender (0=male, 1=female)	0.11	0.07	N/A	.14
		Age (0=age 20)	−0.03	0.02	N/A	.11
		Full-scale IQ (0=IQ score of 110)	0.02	0.01	N/A	.02^d^
	**Session level (level 1)**					
		Subjective high (CC^e^)	−0.01	0.01	N/A	.45
		Distraction (CC)	−0.10	0.03	N/A	.001^b^
		Subjective high (CC) × distraction (CC)	−0.01	0.01	N/A	.23
**Random effects**						
	Level 1 residual variance		0.51	0.01	0.49 to 0.54	N/A
	Intercept		0.06	0.01	0.04 to 0.09	N/A

^a^Not applicable.

^b^*P*<.01.

^c^PM: person mean scores, reflecting individual differences in subjective marijuana high or distraction across all sessions.

^d^*P*<.05.

^e^CC: constant-centered scores (centered at 0), reflecting session-to-session variation in scores or session-specific scores.

**Table 8:** Digit symbol substitution task: multilevel model of marijuana high in relation to number of correct responses.

Effects			DSST^a^ number of correct responses (n=60)			
			Estimate	SE	95% CI	*P* value
**Fixed effects**						
	**Person level (level 2)**					
		Intercept	5.81	9.95	N/A^b^	.56
		Session	0.25	0.02	N/A	.001^c^
		Session^2^ (quadratic)	−0.00	0	N/A	.001^c^
		Subjective high (PM^d^)	−6.83	2.38	N/A	.004^c^
		Distraction (PM)	18.78	7.66	N/A	.01^e^
		Subjective high (PM) × distraction (PM)	4.62	1.81	N/A	.01^e^
		Gender (0=male, 1=female)	−0.23	1.34	N/A	.86
		Age (0=age 20)	−0.40	0.38	N/A	.29
		Full-scale IQ (0=IQ score of 110)	−0.09	0.13	N/A	.47
	**Session level (level 1)**					
		Subjective high (CC^f^)	−0.18	0.07	N/A	.01^e^
		Distraction (CC)	−0.86	0.22	N/A	.001^c^
		Subjective high (CC) × distraction (CC)	0.08	0.04	N/A	.07
**Random effects**						
	Level 1 residual variance		24.66	0.7	23.33 to 26.08	N/A
	Intercept		23.21	4.37	16.04 to 33.57	N/A

^a^DSST: digit symbol substitution task.

^b^Not applicable.

^c^*P*<.01.

^d^PM: person mean scores, reflecting individual differences in subjective marijuana high or distraction across all sessions.

^e^*P*<.05.

^f^CC: constant-centered scores (centered at 0), reflecting session-to-session variation in scores or session-specific scores.

For all 3 tasks, the effect of increasing session-level distraction was significantly associated with fewer correct responses: Flowers task (B=−0.13; SE 0.03; *P*=.001), marijuana Stroop (B=−0.10; SE 0.03; *P*=.001), and DSST (B=−0.86; SE 0.22; *P*=.001). The association of distraction person mean with the number of correct responses was significant for marijuana Stroop (B=−0.82; SE 0.41; *P*=.046) and DSST (B=18.78; SE 7.66; *P*=.01) but in opposite directions. Specifically, for marijuana Stroop, increasing distraction person mean had a unique association with fewer correct responses (controlling for a specific occasion’s rating of distraction), whereas for DSST, increasing distraction person mean predicted an increase in the number of correct DSST responses (over and above a given session’s rating of distraction).

**Figure 6:**
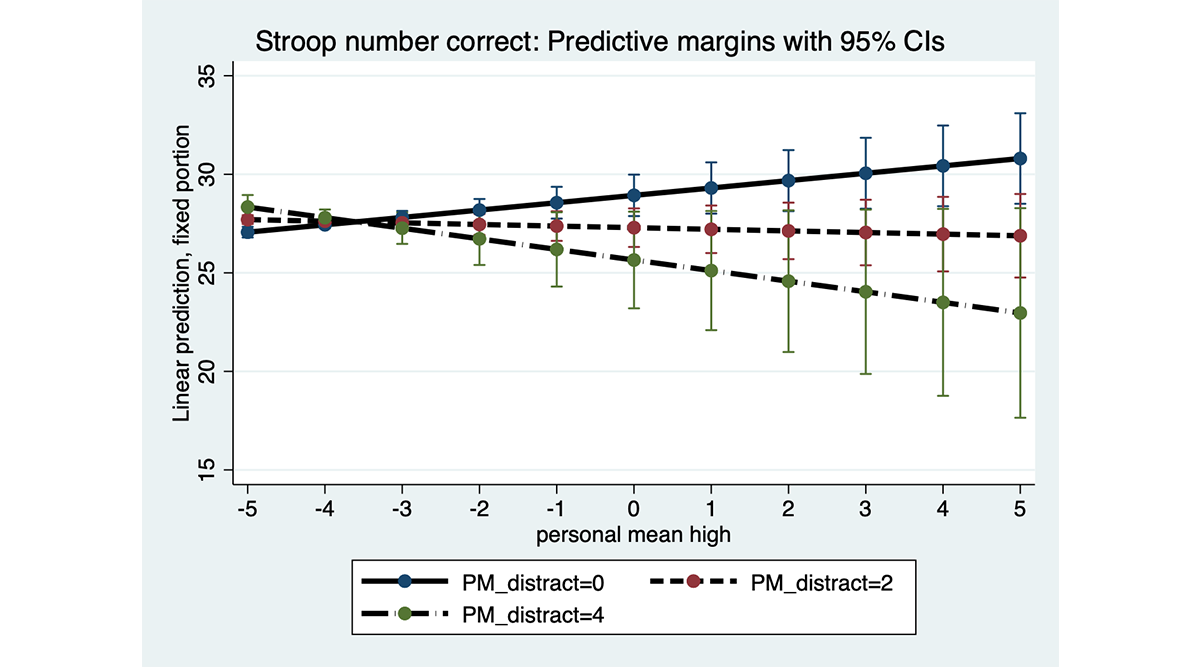
Marijuana Stroop: distraction person mean (PM) as a moderator of the association between subjective high PM and number of correct responses. Distraction PM at low (0), moderate (2) and high levels (4). PM: person mean.

**Figure 7:**
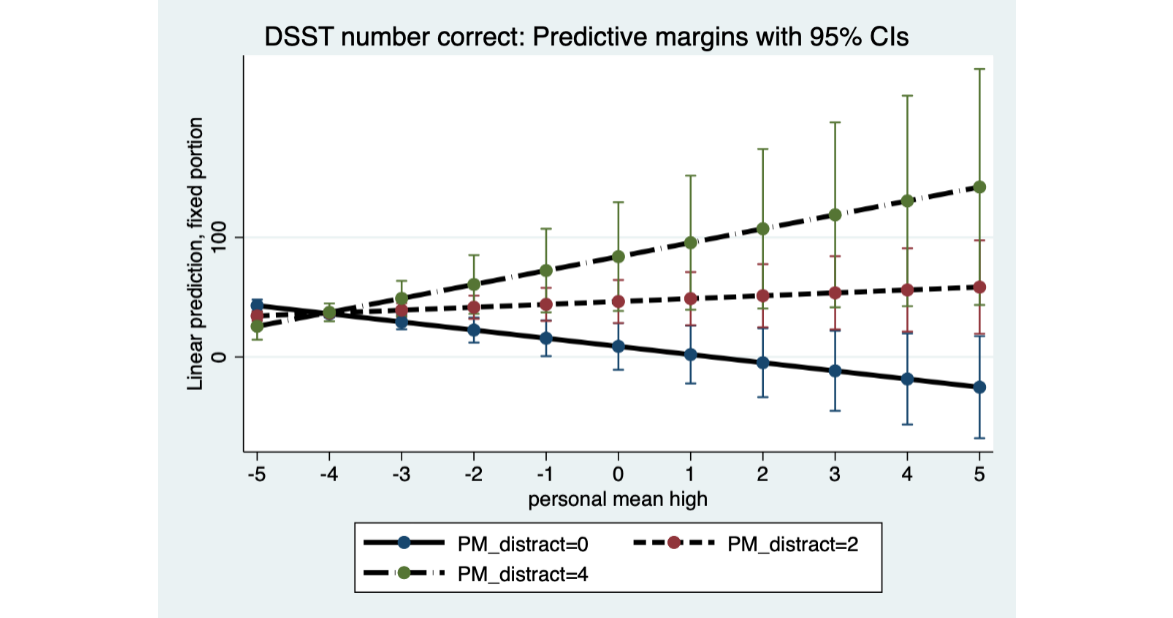
Digit symbol substitution task: distraction person mean (PM) as a moderator of the association between subjective high PM and number of correct responses. Distraction PM at low (0), moderate (2), and high levels (4). DSST: digit symbol substitution task; PM: person mean.

As found for reaction time, in number of correct responses for both marijuana Stroop and DSST, there was a significant interaction of subjective marijuana high person mean with distraction person mean: marijuana Stroop (B=−0.22; SE 0.10; *P*=.02) and DSST (B=4.62; SE 1.81; *P*=.01). For both tasks (Figures 6 and 7), when the subjective marijuana high person mean was on average low, number of correct responses was estimated to be similar across levels of the distraction person mean. When the average level of distraction person mean was high, however, number of correct responses for the marijuana Stroop was estimated to decrease with increasing subjective high person mean. In contrast, for DSST and contrary to the hypothesis, when the average level of distraction person mean was high, number of correct responses for DSST was estimated to show some increase as the average subjective marijuana high person mean increased (although the 95% CI was wide at the highest levels of contextual distraction).

## Discussion

### Principal Findings

Young adults in this pilot study reported, on average, a *moderate* level of subjective marijuana high when using marijuana (mean 4.7; range 1-10), and roughly one distraction, on average, when completing brief mobile cognitive tasks in the natural environment. For all 3 mobile cognitive tasks, as the average rating of subjective marijuana high increased, average reaction time showed a statistically significant increase, suggesting that the mobile tasks were sensitive to psychomotor slowing associated with acute marijuana use in the natural environment, above and beyond practice effects. The statistically significant acute effects of marijuana in relation to reaction time were small, in the context of average *moderate* ratings of subjective marijuana high. Furthermore, for the Flowers task and DSST, individuals with a greater average rating of subjective marijuana high had, at a statistically significant level, slightly fewer correct responses, compared with those with lower average subjective marijuana high ratings, further suggesting sensitivity of these 2 mobile tasks to acute marijuana effects collected *in the wild*, as well as some individual differences in marijuana effects. In the uncontrolled daily life testing situations in which the mobile tasks were completed, distraction ratings were uniquely, significantly associated with only certain aspects of task performance (eg, number of correct responses, controlling for covariates) and also significantly moderated the association between ratings of subjective marijuana high and task performance, albeit in ways that were sometimes contrary to prediction, and depended on specific task characteristics in this pilot study.

A consistent finding across the mobile tasks was that, on average, an increasing rating of subjective marijuana high was significantly associated with slower average reaction time. Although other studies have shown slower reaction time for mobile cigarette smoking Stroop [22,23], this is the first study, to our knowledge, to show significantly slower reaction time for a mobile marijuana Stroop in relation to a numerically rated measure (0-10 scale) of subjective marijuana high. Importantly, ratings of subjective marijuana high showed a small, significant correlation with the number of hits, providing some support for the validity of this study’s subjective marijuana high measure. However, there was no correlation between reports of grams consumed and subjective marijuana high. The absence of a correlation between the quantity reported in grams and the rating of subjective marijuana high suggests individual differences in tolerance to marijuana. Notably, the assessment of self-reported marijuana quantity is challenging [37] and warrants further study, with previous mobile cognitive assessment limited to only reporting any marijuana use (yes or no) [18].

Results also indicated, for the marijuana Stroop task and DSST, that greater the average rating of subjective marijuana high, slower the response time, at a statistically significant level, after controlling for that session’s rating of subjective marijuana high. Thus, a person’s typical level of marijuana use appears to have an effect on response time to these mobile cognitive tasks over and above ratings of acute subjective marijuana high at each session, suggesting possible unique effects of, for example, a person’s pattern of chronic marijuana use on a specific indicator of task performance [6].

Variations in the test environment, such as distractions, can influence task performance [26]. The slightly lower average number of distractions reported when high (vs not high), and very small negative correlation (*r*=−0.04; *P*=.046) between the number of distractions and subjective marijuana high ratings, might reflect that some individuals use marijuana specifically to take a break (eg, *relax*) from distractions in the environment. Alternatively, acute effects of marijuana might reduce the awareness of peripheral distractions for some individuals in certain contexts. The effect of a person’s average level of distraction on number of correct responses was significant for the marijuana Stroop task and DSST, but in opposite directions. This finding suggests the importance of considering how task demand characteristics, such as task complexity, and other factors (eg, motivation, effort), including improved measurement of distraction (ie, multi-item self-report and objective measure), are associated with mobile task performance.

Significant interactions of subjective marijuana high and distraction were found only for the marijuana Stroop task and DSST, for both reaction time and number of correct responses, in this pilot study. For the reaction time outcome, both the marijuana Stroop task and DSST showed a similar pattern of results for the distraction interaction. Specifically, as predicted, and consistent with acute effects of marijuana use on slowing of psychomotor functioning, a person who reported a low average level of distraction had slower reaction time as the average subjective marijuana high rating increased. However, contrary to prediction a person who reported a high average level of distraction was estimated (with wide CIs, suggesting a cautious interpretation) to have *faster* reaction time on these 2 tasks as the average subjective marijuana high rating increased. Faster reaction time suggests the possibility of impulsive responding for some individuals on certain tasks. Regarding the outcome of number of correct responses, results of the distraction interaction differed for the marijuana Stroop task and DSST, and only provided partial support for the hypotheses. Given that the results for some distraction interaction hypotheses were contrary to prediction, interpretation of the distraction interaction results warrants caution due to wide 95% CIs at the highest levels of distraction. The mixed findings for the distraction interactions highlight the need for improved distraction measurement, given self-reporting using a single distraction item.

### Limitations

This pilot study had limitations. On average, this young adult sample was well educated and reported a moderate level of marijuana-related risk, limiting generalizability. Compliance could be improved (eg, no reminders given), and technical issues (eg, app crashes) reduced completion rates. The 5-hour window to complete fixed time assessments accommodated individual schedules but allowed completion (when not high on marijuana) at personally convenient times. Self-reported data (eg, marijuana use start time, subjective high rating, and quantity) are subject to bias (eg, under- or overreporting). Although mobile cognitive tasks were triggered immediately after the phone survey rating of subjective marijuana high, marijuana effects are short-lived [38], cumulative effects of marijuana use on cognitive functioning could be considered [39], and uncontrolled factors (eg, motivation, other substance use and cause, health conditions such as dyslexia) can impact task performance. The single-item measure of distraction was not well defined and might serve as a proxy for unmeasured contextual influences (eg, presence of others and ambient noise), highlighting the need for improved measurement. Flowers and marijuana Stroop tasks showed possible ceiling effects for number of correct responses. Owing to collinearity and small cell sizes for certain substances, the effects of co-occurring substance use (eg, nicotine) on task performance were not examined. The effects of a person’s level of tolerance and cannabis withdrawal on task performance await future research. Correction for multiple comparisons was not done in this pilot study.

### Conclusions

Little is known regarding the real-time cognitive impacts resulting from marijuana use in daily life. Although differences in task performance on the brief mobile cognitive tests when high on marijuana vs not high were small, they were statistically significant and observed for both reaction time and number of correct responses across tasks assessing different cognitive functions. Mobile technology to help detect impacts of acute episodes of marijuana use on cognitive functioning in real time, in the natural environment, could support health care monitoring and provide ongoing feedback to individuals to meet personal health goals [40]. The potential adverse consequences of acute marijuana use on cognitive functioning (eg, while driving) and possible cumulative effects of chronic heavy marijuana use on health compel the development of real-time, mobile methods of monitoring cognitive functioning in the natural environment to help guide personal decision making regarding health behaviors.

## Appendix

Multimedia Appendix 1 Log Likelihood difference testing.

Multimedia Appendix 2 Number of days to complete up to 60 sessions per participant.

Multimedia Appendix 3 Number of completed sessions per participant.

## Abbreviations

CC: constant-centered
DSST: digit symbol substitution task
EMA: ecological momentary assessment
FSIQ: full-scale IQ
ICC: intraclass correlation
NIDA: National Institute on Drug Abuse
OR: odds ratio

## References

[1] Conroy DA, Kurth ME, Brower KJ, Strong DR, Stein MD (2015). Impact of marijuana use on self-rated cognition in young adult men and women. Am J Addict.

[2] Volkow ND, Swanson JM, Evins AE, DeLisi LE, Meier MH, Gonzalez R, Bloomfield MA, Curran HV, Baler R (2016). Effects of cannabis use on human behavior, including cognition, motivation, and psychosis: a review. JAMA Psychiatry.

[3] Phillips KT, Phillips MM, Lalonde TL, Tormohlen KN (2015). Marijuana use, craving, and academic motivation and performance among college students: an in-the-moment study. Addict Behav.

[4] Huestis MA (2015). Cannabis-impaired driving: a public health and safety concern. Clin Chem.

[5] The National Academies of Science, Engineering, and Medicine, Committee on the Health Effects of Marijuana: An Evidence Review and Research, Board on Population Health and Public Health Practice, Health and Medicine Division (2017). The Health Effects of Cannabis and Cannabinoids: The Current State of Evidence and Recommendations for Research.

[6] Scott JC, Slomiak ST, Jones JD, Rosen AF, Moore TM, Gur RC (2018). Association of cannabis with cognitive functioning in adolescents and young adults: a systematic review and meta-analysis. JAMA Psychiatry.

[7] Zuurman L, Ippel AE, Moin E, van Gerven JM (2009). Biomarkers for the effects of cannabis and THC in healthy volunteers. Br J Clin Pharmacol.

[8] Crane NA, Schuster RM, Fusar-Poli P, Gonzalez R (2013). Effects of cannabis on neurocognitive functioning: recent advances, neurodevelopmental influences, and sex differences. Neuropsychol Rev.

[9] Schreiner AM, Dunn ME (2012). Residual effects of cannabis use on neurocognitive performance after prolonged abstinence: a meta-analysis. Exp Clin Psychopharmacol.

[10] Crean RD, Tapert SF, Minassian A, Macdonald K, Crane NA, Mason BJ (2011). Effects of chronic, heavy cannabis use on executive functions. J Addict Med.

[11] Bot BM, Suver C, Neto EC, Kellen M, Klein A, Bare C, Doerr M, Pratap A, Wilbanks J, Dorsey ER, Friend SH, Trister AD (2016). The mPower study, Parkinson disease mobile data collected using ResearchKit. Sci Data.

[12] Field M (2005). Cannabis 'dependence' and attentional bias for cannabis-related words. Behav Pharmacol.

[13] Bettcher BM, Libon DJ, Kaplan E, Swenson R, Penney DL, Kreutzer JS, DeLuca J, Caplan B (2011). Digit symbol substitution test. Encyclopedia of Clinical Neuropsychology.

[14] Cameron E, Sinclair W, Tiplady B (2001). Validity and sensitivity of a pen computer battery of performance tests. J Psychopharmacol.

[15] Chaytor N, Schmitter-Edgecombe M (2003). The ecological validity of neuropsychological tests: a review of the literature on everyday cognitive skills. Neuropsychol Rev.

[16] Sliwinski MJ, Mogle JA, Hyun J, Munoz E, Smyth JM, Lipton RB (2018). Reliability and validity of ambulatory cognitive assessments. Assessment.

[17] Moore RC, Swendsen J, Depp CA (2017). Applications for self-administered mobile cognitive assessments in clinical research: a systematic review. Int J Methods Psychiatr Res.

[18] Schuster RM, Mermelstein RJ, Hedeker D (2016). Ecological momentary assessment of working memory under conditions of simultaneous marijuana and tobacco use. Addiction.

[19] Zhang MW, Ying J, Wing T, Song G, Fung DS, Smith HE (2018). Cognitive biases in cannabis, opioid, and stimulant disorders: a systematic review. Front Psychiatry.

[20] Field M, Munafò MR, Franken IHA (2009). A meta-analytic investigation of the relationship between attentional bias and subjective craving in substance abuse. Psychol Bull.

[21] Spanakis P, Jones A, Field M, Christiansen P (2019). A Stroop in the hand is worth two on the laptop: superior reliability of a smartphone based alcohol Stroop in the real world. Subst Use Misuse.

[22] Waters AJ, Li Y (2008). Evaluating the utility of administering a reaction time task in an ecological momentary assessment study. Psychopharmacology (Berl).

[23] Waters AJ, Szeto EH, Wetter DW, Cinciripini PM, Robinson JD, Li Y (2014). Cognition and craving during smoking cessation: an ecological momentary assessment study. Nicotine Tob Res.

[24] Bouvard A, Dupuy M, Schweitzer P, Revranche M, Fatseas M, Serre F, Misdrahi D, Auriacombe M, Swendsen J (2018). Feasibility and validity of mobile cognitive testing in patients with substance use disorders and healthy controls. Am J Addict.

[25] Pal R, Mendelson J, Clavier O, Baggott MJ, Coyle J, Galloway GP (2016). Development and testing of a smartphone-based cognitive/neuropsychological evaluation system for substance abusers. J Psychoactive Drugs.

[26] Prince J, Arora S, de Vos M (2018). Big data in Parkinson's disease: using smartphones to remotely detect longitudinal disease phenotypes. Physiol Meas.

[27] AWARE Framework.

[28] NuRelm.

[29] Blair JR, Spreen O (1989). Predicting premorbid IQ: a revision of the national adult reading test. Clin Neuropsychol.

[30] NIDA National Institute on Drug Abuse (NIDA).

[31] Bright P, Hale E, Gooch VJ, Myhill T, van der Linde I (2018). The National Adult Reading Test: restandardisation against the Wechsler Adult Intelligence Scale-Fourth edition. Neuropsychol Rehabil.

[32] Kay M, Rector K, Consolvo S, Greenstein B, Wobbrock J, Watson N, Kientz J (2013). PVT-Touch: Adapting a Reaction Time Test for Touchscreen Devices. Proceedings of the 2013 7th International Conference on Pervasive Computing Technologies for Healthcare and Workshops.

[33] Waters AJ, Marhe R, Franken IH (2012). Attentional bias to drug cues is elevated before and during temptations to use heroin and cocaine. Psychopharmacology (Berl).

[34] Hallgren KA, McCrady BS (2013). Interference in the alcohol Stroop task with college student binge drinkers. J Behav Health.

[35] StataCorp (2017). Stata Statistical Software: Release 15.

[36] Hoffman L (2015). Longitudinal Analysis: Modeling Within-Person Fluctuation and Change.

[37] Prince MA, Conner BT, Pearson MR (2018). Quantifying cannabis: a field study of marijuana quantity estimation. Psychol Addict Behav.

[38] Riedel G, Davies SN (2005). Cannabinoid function in learning, memory and plasticity. Handb Exp Pharmacol.

[39] Pope HG, Gruber AJ, Yurgelun-Todd D (2001). Residual neuropsychologic effects of cannabis. Curr Psychiatry Rep.

[40] Abdullah S, Murnane EL, Matthews M, Kay M, Kientz JA, Gay G, Choudhury T (2016). Cognitive Rhythms: Unobtrusive and Continuous Sensing of Alertness Using a Mobile Phone. Proceedings of the 2016 ACM International Joint Conference.

