# Supplementary material for: Mobile Assessment of Acute Effects of Marijuana on Cognitive Functioning in Young Adults: Observational Study
Source: JMIR Mhealth Uhealth. 2020 Mar 10;8(3):e16240. doi: 10.2196/16240 (PMC7093776; doi:10.2196/16240)

Supplementary materials

Log Likelihood difference testing

Table S1. Flowers multilevel model of marijuana high in relation to reaction time (n=58)

| Fixed Effects | Subjective High Only | | | Subjective High and Distract | | |
| --- | --- | --- | --- | --- | --- | --- |
| **Person Level (Level 2)** | **Estimate** | **SE** | **P** | **Estimate** | **SE** | **P** |
| Intercept | 493.34 | 51.70 | .001 | 476.72 | 53.46 | .001 |
| Session | -.13 | .08 | .101 | -.14 | .08 | .084 |
| Session^2^ (quadratic) |  |  |  |  |  |  |
| Subjective High (PM) | 32.97 | 11.92 | .006 | 30.35 | 11.96 | .011 |
| Distraction (PM) |  |  |  | 7.33 | 9.97 | .462 |
| Gender  (0=Male, 1=Female) | -12.80 | 10.51 | .223 | -11.72 | 10.54 | .266 |
| Age (0= age 20) | 6.64 | 3.07 | .031 | 7.15 | 3.08 | .020 |
| Full Scale IQ  (0= IQ score of 110) | 2.31 | .90 | .010 | 2.04 | .93 | .029 |
| **Session Level (Level 1)** | **Estimate** | **SE** | **P** | **Estimate** | **SE** | **P** |
| Subjective High (CC) | 1.82 | .62 | .004 | 1.88 | .62 | .003 |
| Distraction (CC) |  |  |  | -2.69 | 1.63 | .099 |
| **Random Effects** | **Estimate** | **SE** | **95% CI** | **Estimate** | **SE** | **95% CI** |
| Level 1 residual variance | 3549.76 | 100.58 | 3358.01, 3752.47 | 3417.46 | 99.96 | 3227.05, 3619.11 |
| Intercept | 1340.23 | 269.86 | 903.20, 1988.71 | 1295.71 | 282.89 | 844.63,  1987.69 |
| Distraction |  |  |  | 55.44 | 28.19 | 20.47, 150.19 |
| Covariance (intercept, distraction) |  |  |  | -11.43 | 72.61 | -153.73, 130.88 |
| **Model Difference Testing/Fit Criteria** | | | |  | | |
| -2LL | -28243.12 |  | | -27053.04 | Including random effect of distraction improves model fit (lower AIC, BIC); significance testing not used because number of observations differ | |
| AIC | 28261.12 |  | | 27079.05 |  |  |
| BIC | 28279.67 |  |  | 27105.83 |  |  |

Notes: CC=Constant centered scores (centered at 0), reflecting session-specific scores or session-to-session variation in scores

PM=person mean scores, reflecting individual differences in subjective marijuana high or distraction across all sessions. SE=Standard Error

| **Interaction: High x Distract** | | |
| --- | --- | --- |
| **-2LL Model Difference Testing and Fit Criteria** | | |
| -2LL | -27051.58 | P=.482, no significant difference of model with interaction relative to main effects only (“Subjective High and Distract Model”) |
| AIC | 27081.59 |  |
| BIC | 27112.49 |  |

-2LL= -2 Log Likelihood, AIC=Akaike Information Criterion, BIC=Bayesian Information Criterion

Table S2. Stroop multilevel model of marijuana high in relation to reaction time (n=60)

| Fixed Effects | Subjective High Only | | | Subjective High and Distract | | |
| --- | --- | --- | --- | --- | --- | --- |
| **Person Level (Level 2)** | **Estimate** | **SE** | **P** | **Estimate** | **SE** | **P** |
| Intercept | 791.07 | 68.64 | .001 | 778.29 | 67.88 | .001 |
| Session | -.54 | .14 | .001 | -.60 | .10 | .001 |
| Session^2^ (quadratic) |  |  |  |  |  |  |
| Subjective High (PM) | 19.59 | 15.94 | .219 | 19.93 | 15.47 | .198 |
| Distraction (PM) |  |  |  | 8.87 | 14.19 | .532 |
| Gender  (0=Male, 1=Female) | 5.41 | 15.57 | .728 | -2.46 | 15.10 | .870 |
| Age (0= age 20) | 13.81 | 4.48 | .002 | 13.63 | 4.30 | .002 |
| Full Scale IQ  (0= IQ score of 110) | -.53 | 1.35 | .696 | -1.58 | 1.36 | .244 |
| **Session Level (Level 1)** | **Estimate** | **SE** | **P** | **Estimate** | **SE** | **P** |
| Subjective High (CC) | 2.02 | 1.06 | .057 | 1.38 | .80 | .082 |
| Distraction (CC) |  |  |  | 5.05 | 2.51 | .044 |
| **Random Effects** | **Estimate** | **SE** | **95% CI** | **Estimate** | **SE** | **95% CI** |
| Level 1 residual variance | 11006.19 | 306.12 | 10422.26, 11622.84 | 5865.79 | 168.54 | 5544.58, 6205.61 |
| Intercept | 2964.47 | 601.98 | 1991.12, 4413.65 | 3183.09 | 669.55 | 2107.66, 4807.25 |
| Distraction |  |  |  | 191.36 | 69.02 | 94.37, 388.02 |
| Covariance (intercept, distraction) |  |  |  | -262.95 | 175.96 | -607.82, 81.93 |
| **Model Difference Testing/Fit Criteria** | | | |  |  |  |
| -2LL | -32281.72 |  |  | -29483.18 | Random effect of distraction is significant, P=.001 | |
| AIC | 32299.72 |  |  | 29509.18 |  | |
| BIC | 32318.57 |  |  | 29536.41 |  |  |

| **Interaction: High x Distract** | | |
| --- | --- | --- |
| **-2LL Model Difference Testing and Fit Criteria** | | |
| -2LL | -29473.00 | P=.006, significantly better fit of model with interaction relative to main effects only (“Subjective High and Distract Model”) |
| AIC | 29503.01 |  |
| BIC | 29534.43 |  |

Note: abbreviations as in Table S1

Table S3. DSST multilevel model of marijuana high in relation to reaction time (n=60)

| Fixed Effects | Subjective High Only | | | Subjective High and Distract | | |
| --- | --- | --- | --- | --- | --- | --- |
| **Person Level (Level 2)** | **Estimate** | **SE** | **P** | **Estimate** | **SE** | **P** |
| Intercept | 1841.75 | 144.33 | .001 | 1853.59 | 147.28 | .001 |
| Session | -9.18 | .47 | .001 | -9.46 | .46 | .001 |
| Session^2^ (quadratic) | .11 | .01 | .001 | .12 | .01 | .001 |
| Subjective High (PM) | 59.02 | 33.48 | .078 | 64.91 | 33.58 | .053 |
| Distraction (PM) |  |  |  | 13.31 | 30.88 | .666 |
| Gender  (0=Male, 1=Female) | -1.12 | 32.77 | .973 | -11.51 | 33.05 | .728 |
| Age (0= age 20) | 9.43 | 9.32 | .312 | 7.63 | 9.34 | .414 |
| Full Scale IQ  (0= IQ score of 110) | 2.82 | 2.80 | .313 | 1.82 | 2.95 | .537 |
| **Session Level (Level 1)** | **Estimate** | **SE** | **P** | **Estimate** | **SE** | **P** |
| Subjective High (CC) | 1.84 | 1.07 | .085 | 2.02 | 1.03 | .050 |
| Distraction (CC) |  |  |  | 6.32 | 2.38 | .008 |
| **Random Effects** | **Estimate** | **SE** | **95% CI** | **Estimate** | **SE** | **95% CI** |
| Level 1 residual variance | 11016.60 | 306.48 | 10431.99, 11633.97 | 9959.67 | 286.10 | 9414.42, 10536.49 |
| Intercept | 14105.29 | 2631.70 | 9785.18, 20332.73 | 14842.09 | 2852.08 | 10184.19, 21630.34 |
| Distraction |  |  |  | 59.06 | 58.44 | 8.49, 410.74 |
| Covariance (intercept, distraction) |  |  |  | -284.01 | 349.54 | -969.11, 401.08 |
| **Model Difference Testing/Fit Criteria** | | | |  |  |  |
| -2LL | -32348.64 |  |  | -30832.64 | Random effect of distraction is ns (P=.422) | |
| AIC | 32368.63 |  |  | 30860.64 |  |  |
| BIC | 32389.58 |  |  | 30889.96 |  |  |

| **Interaction: High x Distract** | | |
| --- | --- | --- |
| **-2LL Model Difference Testing and Fit Criteria** | | |
| -2LL | -30825.50 | P=.028, significantly better fit of model with interaction relative to main effects only (“Subjective High and Distract Model”) |
| AIC | 30857.50 |  |
| BIC | 30891.01 |  |

Note: abbreviations as in Table S1

Table S4. Flowers multilevel model of marijuana high in relation to **number correct** (n=60)

| Fixed Effects | Subjective High Only | | | Subjective High and Distract | | |
| --- | --- | --- | --- | --- | --- | --- |
| **Person Level (Level 2)** | **Estimate** | **SE** | **P** | **Estimate** | **SE** | **P** |
| Intercept | 3.80 | .52 | .001 | 3.76 | .52 | .001 |
| Session | .00 | .00 | .164 | .00 | .00 | .008 |
| Session^2^ (quadratic) |  |  |  |  |  |  |
| Subjective High (PM) | -.13 | .12 | .280 | -.16 | .12 | .185 |
| Distraction (PM) |  |  |  | .06 | .11 | .560 |
| Gender  (0=Male, 1=Female) | -.11 | .12 | .336 | -.04 | .12 | .715 |
| Age (0= age 20) | -.15 | .03 | .001 | -.14 | .03 | .001 |
| Full Scale IQ  (0= IQ score of 110) | .00 | .01 | .721 | .01 | .01 | .338 |
| **Session Level (Level 1)** | **Estimate** | **SE** | **P** | **Estimate** | **SE** | **P** |
| Subjective High (CC) | -.02 | .01 | .034 | -.02 | .01 | .020 |
| Distraction (CC) |  |  |  | -.17 | .02 | .001 |
| **Random Effects** | **Estimate** | **SE** | **95% CI** | **Estimate** | **SE** | **95% CI** |
| Level 1 residual variance | .69 | .02 | .66, .73 | .59 | .02 | .56, .63 |
| Intercept | .17 | .04 | .11, .26 | .16 | .03 | .11, .25 |
| **Model Difference Testing/Fit Criteria** | | | |  |  |  |
| -2LL | -6688.60 |  |  | -6035.10 | Based on BIC (significance testing not used because number of observations differ), this model’s fit is better relative to subjective high only model | |
| AIC | 6706.60 |  |  | 6057.10 |  |  |
| BIC | 6725.45 |  |  | 6080.14 |  |  |

| Interaction: High x Distract | | |
| --- | --- | --- |
| -2LL Model Difference Testing and Fit Criteria | | |
| -2LL | -6032.02 | P=.215, not significantly better fit of model with interaction relative to main effects only (“Subjective High and Distract Model”) |
| AIC | 6058.02 |  |
| BIC | 6085.25 |  |

Note: abbreviations as in Table S1

Table S5. Stroop multilevel model of marijuana high in relation to number correct (n=60)

| Fixed Effects | Subjective High Only | | | Subjective High and Distract | | |
| --- | --- | --- | --- | --- | --- | --- |
| **Person Level (Level 2)** | **Estimate** | **SE** | **P** | **Estimate** | **SE** | **P** |
| Intercept | 28.06 | .33 | .001 | 27.99 | .34 | .001 |
| Session | -.00 | .00 | .001 | -.00 | .00 | .001 |
| Session^2^ (quadratic) |  |  |  |  |  |  |
| Subjective High (PM) | .13 | .08 | .097 | .13 | .08 | .097 |
| Distraction (PM) |  |  |  | .13 | .07 | .070 |
| Gender  (0=Male, 1=Female) | .10 | .08 | .208 | .09 | .08 | .221 |
| Age (0= age 20) | -.03 | .02 | .149 | -.03 | .02 | .164 |
| Full Scale IQ  (0= IQ score of 110) | .01 | .01 | .058 | .01 | .01 | .134 |
| **Session Level (Level 1)** | **Estimate** | **SE** | **P** | **Estimate** | **SE** | **P** |
| Subjective High (CC) | -.01 | .01 | .047 | -.02 | .01 | .032 |
| Distraction (CC) |  |  |  | -.07 | .02 | .001 |
| **Random Effects** | **Estimate** | **SE** | **95% CI** | **Estimate** | **SE** | **95% CI** |
| Level 1 residual variance | .51 | .01 | .49, .54 | .51 | .01 | .49, .54 |
| Intercept | .06 | .01 | .04, .10 | .06 | .01 | .04, .10 |
| **Model Difference Testing/Fit Criteria** | | | |  |  |  |
| -2LL | -5858.62 |  |  | -5629.86 | Random effects of subjective high and distraction were not significant. | |
| AIC | 5876.62 |  |  | 5651.86 |  |  |
| BIC | 5895.47 |  |  | 5674.89 |  |  |

| **Interaction: High x Distract** | | |
| --- | --- | --- |
| **-2LL Model Difference Testing and Fit Criteria** | | |
| -2LL | -5622.82 | P=.030, significantly better fit of model with interaction relative to main effects only (“Subjective High and Distract Model”) |
| AIC | 5648.82 |  |
| BIC | 5676.04 |  |

Note: abbreviations as in Table S1

Table S6. DSST multilevel model of marijuana high in relation to **number correct** (n=60)

| Fixed Effects | Subjective High Only | | | Subjective High and Distract | | |
| --- | --- | --- | --- | --- | --- | --- |
| **Person Level (Level 2)** | **Estimate** | **SE** | **P** | **Estimate** | **SE** | **P** |
| Intercept | 26.32 | 6.36 | .001 | 26.52 | 6.31 | .001 |
| Time | .22 | .02 | .001 | .25 | .02 | .001 |
| Time^2^ (quadratic) | -.00 | .00 | .001 | -.00 | .00 | .001 |
| Subjective High (PM) | -1.58 | 1.48 | .285 | -1.84 | 1.44 | .200 |
| Distraction (PM) |  |  |  | -.50 | 1.32 | .705 |
| Gender  (0=Male, 1=Female) | -.69 | 1.44 | .632 | .05 | 1.42 | .971 |
| Age (0= age 20) | -.53 | .41 | .197 | -.47 | .40 | .242 |
| Full Scale IQ  (0= IQ score of 110) | -.04 | .12 | .726 | .03 | .13 | .802 |
| **Session Level (Level 1)** | **Estimate** | **SE** | **P** | **Estimate** | **SE** | **P** |
| Subjective High (CC) | -.07 | .06 | .229 | -.09 | .05 | .082 |
| Distraction (CC) |  |  |  | -1.21 | .10 | .001 |
| **Random Effects** | **Estimate** | **SE** | **95% CI** | **Estimate** | **SE** | **95% CI** |
| Level 1 residual variance | 30.85 | .86 | 29.21, 32.58 | 24.70 | .70 | 23.36, 26.11 |
| Intercept | 27.17 | 5.12 | 18.79, 39.31 | 25.92 | 4.86 | 17.94, 37.45 |
| **Model Difference Testing/Fit Criteria** | | | |  |  |  |
| -2LL | -16797.78 |  |  | -15582.30 | Random effects of subjective high and distraction were not significant. | |
| AIC | 16817.78 |  |  | 15606.29 |  |  |
| BIC | 16838.73 |  |  | 15631.42 |  |  |

| **Interaction: High x Distract** | | |
| --- | --- | --- |
| **-2LL Model Difference Testing and Fit Criteria** | | |
| -2LL | -15572.66 | P=.008, significantly better fit of model with interaction relative to main effects only (“Subjective High and Distract Model”) |
| AIC | 15600.67 |  |
| BIC | 15629.99 |  |

Note: abbreviations as in Table S1

Supplemental Table S7

Mobile session completion (N=2,703) by Day of Week and Time of Day

|  | **Sun** | **Mon** | **Tues** | **Wed** | **Thu** | **Fri** | **Sat** | **Total** |
| --- | --- | --- | --- | --- | --- | --- | --- | --- |
| **Morning**  6am-11:59am | 67 | 68 | 72 | 81 | 80 | 84 | 68 | 520 |
| **Afternoon**  12pm-5:59pm | 151 | 141 | 119 | 138 | 140 | 152 | 147 | 988 |
| **Evening**  6pm-11:59pm | 126 | 142 | 155 | 145 | 169 | 155 | 131 | 1023 |
| **Midnight-early am**  12am-5:59am | 32 | 15 | 31 | 23 | 18 | 28 | 25 | 172 |
| **Total** | 376 | 366 | 377 | 387 | 407 | 419 | 371 | 2703 |

Supplemental Table S8

Mobile session completion for reports subjective rating of marijuana high >0 (N=451) by Day of Week and Time of Day

|  | **Sun** | **Mon** | **Tues** | **Wed** | **Thu** | **Fri** | **Sat** | **Total** |
| --- | --- | --- | --- | --- | --- | --- | --- | --- |
| **Morning**  6am-11:59am | 2 | 3 | 4 | 8 | 5 | 10 | 4 | 36 |
| **Afternoon**  12pm-5:59pm | 12 | 5 | 5 | 7 | 9 | 11 | 8 | 57 |
| **Evening**  6pm-11:59pm | 26 | 28 | 31 | 36 | 42 | 26 | 23 | 212 |
| **Midnight-early am**  12am-5:59am | 28 | 14 | 23 | 21 | 14 | 26 | 20 | 146 |
| **Total** | 68 | 50 | 63 | 72 | 70 | 73 | 55 | 451 |

Supplementary Figure 1


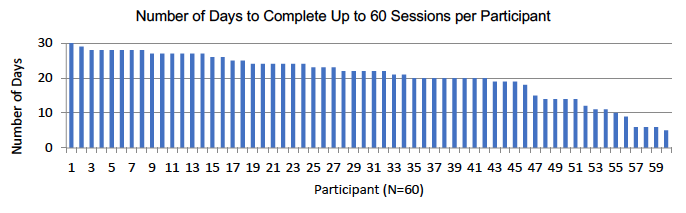


Supplementary Figure 2


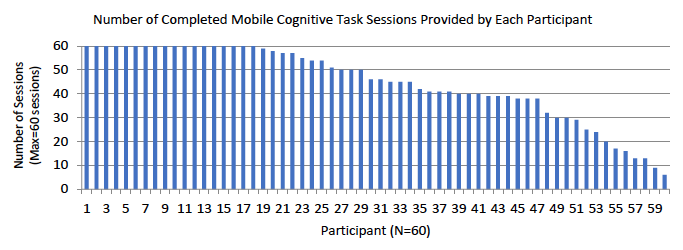

Supplement: Multimedia Appendix 1 [file mhealth_v8i3e16240_app1.docx]
