# Supplementary figures and images for: Mobile Assessment of Acute Effects of Marijuana on Cognitive Functioning in Young Adults: Observational Study
Source: JMIR Mhealth Uhealth. 2020 Mar 10;8(3):e16240. doi: 10.2196/16240 (PMC7093776; doi:10.2196/16240)

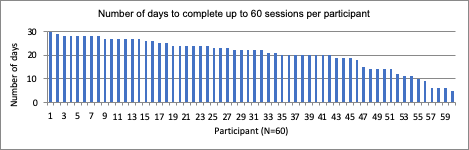

Supplement: Multimedia Appendix 2 [file mhealth_v8i3e16240_app2.png]

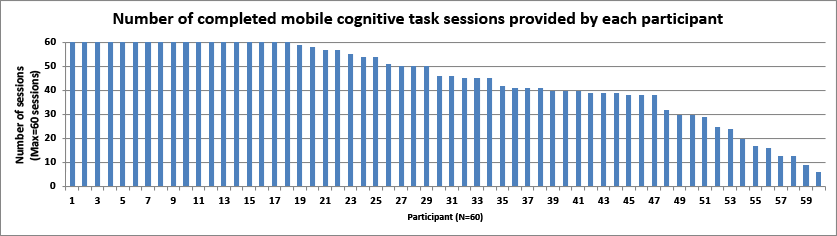

Supplement: Multimedia Appendix 3 [file mhealth_v8i3e16240_app3.png]
